# Removal of FIGO V and VI fibroids with a combined size greater than 5 cm quadruples spontaneous fecundity relative to myomectomy for those with smaller fibroids

**DOI:** 10.1007/s00404-025-08063-x

**Published:** 2025-05-23

**Authors:** Magdalena Boegl, Johannes Ott, Elena Seidl, Tal Goldstein, John Preston Parry, Marlene Hager

**Affiliations:** 1 Clinical Division of Gynecological Endocrinology and Reproductive Medicine, Department of Obstetrics and Gynecology, Medical University of Vienna, Spitalgasse 23, 1090 Vienna, Austria; 2 Positive Steps Fertility, Madison, MS USA; 3 Department of Obstetrics and Gynecology, University of Mississippi Medical Center, Jackson, MS USA

**Keywords:** Fibroids, FIGO V, FIGO VI, Myomectomy, Pregnancy, Infertility

## Abstract

**Purpose:**

Fibroids are the most common gynecological pathology in reproductive aged women and contribute to 2–3% of infertility cases. After hysteroscopic removal of submucosal FIGO 0 and I fibroids, pregnancy rates of 60% to 90% can be achieved. Pregnancy rates after non-hysteroscopic removal of subserosal FIGO V and VI fibroids remain controversial.

**Methods:**

We examined all myomectomies per laparoscopy/laparotomy for FIGO V and VI fibroids performed at the Clinical Division of Gynecological Endocrinology and Reproductive Medicine, Medical University of Vienna, from 2012 to 2021. All women with primary and secondary infertility between the ages of 18 and 40 years with 1–3 subserous fibroids without additionally identified causes for infertility were included. The outcome was the clinical pregnancy rate within 12 months after a postoperative non-conception window. A logistic regression model was used to assess associations between patient characteristics and postoperative pregnancy rates. The association was estimated as odds ratio (OR) with the respective 95% confidence interval (CI).

**Results:**

We included a total of 80 women with a median age of 34.5 years (IQR, 31.4–37.8). Of those, 42 patients (52.5%) had primary infertility and 38 patients (47.5%) had secondary infertility. Fibroid size ranged from 2 to 30 cm with a median size of 7.5 cm. Pregnancy occurred in 36 patients (45.0%) at a median of 4 months (IQR 3.0–7.0) after initial postoperative 6 months, where pregnancy was permitted. Age (OR 0.77, 95% CI 0.67–0.88) and fibroid size (OR 1.25; 95% CI 1.072–1.446) were significantly associated with the occurrence of a clinical pregnancy.

**Conclusion:**

In this cohort of infertile women of reproductive age with FIGO V and VI fibroids, almost half became spontaneously pregnant within 12 months after a postoperative non-conception window of myomectomy per laparoscopy/laparotomy. Patients with larger fibroids were more likely to conceive after myomectomy.

## What does this study add to the clinical work

**Table:** 

The effects of subserosal fibroid removal on fertility are still unknown. This observational study found that the removal of FIGO V and VI fibroids with a combined size greater than 5cm quadruples spontaneous conception compared to myomectomy in those with smaller fibroids.	

## Background

Uterine fibroids are the most common gynecological pathology in reproductive aged women with an estimated lifetime prevalence of up to 70% [1, 2]. Although they are benign tumors that often remain asymptomatic, uterine fibroids are a notable cause of recurrent pregnancy loss and contribute to 2–3% of all cases of infertility [3, 4].

Fibroids are classified by the International Federation of Gynecology and Obstetrics (FIGO) [4] into nine types (0–8), depending on the location (submucosal, intramural, subserosal, and transmural) and the degree of intramural or intracavitary protrusion. Submucosal fibroids (FIGO 0-III) are most frequently associated with infertility. Several hypotheses have already been put forward to explain the connection between fibroids and infertility. One hypothesis suggests that the mechanical deformation of the uterine cavity may hinder the transport of germ cells and embryos, subsequently impairing implantation [5, 6]. Another hypothesis posits that fibroids could alter the expression patterns of angiogenic factors (such as basic fibroblast growth factor and platelet-derived growth factor) [7, 8]. Additional hypotheses why patients with fibroids experience fertility problems include inflammation [7, 9] and alterations of the endometrial lining [9]. As published by the American Society for Reproductive Medicine [5], there is a recommendation surgical removal if a desired pregnancy does not occur after all other causes for infertility have been ruled out. Pregnancy rates of up to 60–89.2% can be achieved with hysteroscopic myectomy [10, 11].

In contrast, the association of fibroids with subserosal localization (FIGO V-VI) and infertility is less clear. Consequently, there are no clear recommendations as to whether FIGO V-VI fibroids should be surgically removed to improve fecundity rates due to heterogeneous study designs, inconsistent nomenclature, diversity of leiomyoma, and patient characteristics including superimposed sources for subfertility, such as male subfertility and endometriosis among others. In addition, subserosal fibroids cannot be treated hysteroscopically instead requiring laparoscopic or laparotomic surgery, reflecting greater effort in intervention. Some data suggest that larger fibroids > 2.85 cm [12, 13] or > 3 cm [12] restrict reproduction, while other findings suggest that fibroids < 5 cm do not [14, 15]. Thus, one could hypothesize that only women with larger FIGO V-VI fibroids might benefit from myomectomy in terms of fertility.

Given the fact that there are little data on the chances of pregnancy after surgery for subserosal fibroids [16, 17], we examined the pregnancy rates of all women with at least 1 year of infertility who underwent laparoscopic or open surgery for subserosal fibroids at our clinic in the last 12 years. In addition, the focus was also on parameters influencing clinical pregnancy, first and foremost being fibroid size.

## Methods

### Study design

We conducted a cohort study to investigate the clinical pregnancy rates within 12 months after myomectomy by laparoscopy/laparotomy and a postoperative non-conception window of 6–9 months after the operation in infertile women with FIGO V and FIGO VI fibroids.

### Study setting and participants

The study was performed at the Clinical Division of Gynecological Endocrinology at the Medical University of Vienna, Austria. All women between 18 and 40 years of age with a history of primary or secondary infertility and ≤ 3 subserous FIGO V and VI fibroids who underwent laparoscopy or laparotomy between January 2012 and December 2021, after all other cases of infertility had been ruled out, were eligible for participation (complete case analysis). We chose to not include women with four or more fibroids due to the small number of such patients in our retrospective data set as well as to keep the patient population as consistent as possible. In each of these patients, all fibroids which had been visible with preoperative transvaginal ultrasound were removed during the operation. Infertility was defined as the inability to conceive within 1 year (or 6 months, if older than 35 years), of regular unprotected intercourse. Primary infertility referred to the inability to achieve pregnancy in individuals who had never achieved a pregnancy before, while secondary infertility was deemed when at least one prior pregnancy had been achieved [18]. Women with FIGO I, II, III, IV, and VII fibroids as well as other possible causes of infertility, which included endometriosis, adenomyosis, fallopian tube obstruction, sexually transmitted diseases, ovulatory disorders (hypogonadotropic hypogonadism, polycystic ovary syndrome, functional hypothalamic amenorrhea, and oligo- or anovulation), diminished ovarian reserve, and an abnormal semen analysis of the partner, were excluded.

All operations, either via laparotomy or laparoscopy, were performed under general anesthesia. The techniques had been described previously [19] and had also been used for prospective studies at the Medical University of Vienna [20]. The decision for either laparoscopy or laparotomy was based on the size and numbers of the fibroids as well as on the patients’ and surgeons’ preference.

### Study outcomes

All patients were recommended to avoid pregnancy for 6–9 months after the operation (non-conception window). The primary study outcome was the clinical pregnancy rate within 12 months after surgical recovery once pregnancy was permitted. Clinical pregnancy was defined as a pregnancy confirmed by a heartbeat on ultrasound. In an exploratory data analysis, possible associations between patient and fibroid characteristics and fecundity were investigated.

The following relevant parameters were included: patient’s age at the time of operation; body mass index (BMI); primary versus secondary infertility; the number and the size (maximum diameter) of the fibroids as well as the sum of the maximum diameter of all fibroids; the type of surgery; and the time to conceive (date from which the patient was allowed to become pregnant to positive pregnancy test, in months). In accordance with the data protection regulations applicable in Austria, all information collected was pseudonymized and transferred to a Microsoft Excel (Microsoft Corporation, Redmon, WA, USA) data processing table. All data were retrieved from the AKIM-software (SAP-based patient management system at the Medical University of Vienna).

### Statistical analysis

Categorical variables are presented as absolute numbers (n) and frequencies (%), and continuous variables as median and interquartile range (IQR). For the pregnancy rate, the corresponding 95% CI was calculated. We used a logistic regression model to analyze associations of patient characteristics [including age, body mass index (BMI), and type of infertility] and surgery/fibroid characteristics [including type of myomectomy (laparoscopy versus laparotomy), diameter of the largest fibroid (cm), and the sum of fibroid diameters (cm)] with successful pregnancy. The associations were estimated as odds ratio (OR) with the respective 95% confidence intervals (CI). A two-sided p value < 0.05 was considered statistically significant. The optimal cut-off for fibroid size for the ability to achieve a pregnancy was calculated using receiver-operator characteristic (ROC) curve and was defined at the cut-off value optimizing sensitivity and specificity. For missing data, no data imputation was performed. All outcome data were available for all participants. All statistical analyses were performed using SPSS version 28.0.

This study was approved by the local ethics committee (EK 1271/2023) and conducted in accordance with the Declarations of Helsinki. The graphical abstract was created using PowerPoint by Microsoft Office 365, version 2406.

## Results

A total of 80 women with a median age of 34.6 years (IQR, 31.4–37.8) and a median BMI of 23.7 kg/m^2^ (IQR, 21.7–27.5) were included in the study. About half of women (52.5%, *n* = 42) had primary infertility. Forty-six women (57.5%) had exactly one fibroid, 26.3% (*n* = 21) had two fibroids, and 16% (*n* = 13) had three fibroids. Laparoscopy was performed in the majority of patients (63.7%, *n* = 51), whereas 36.3% (*n* = 29) of patients underwent laparotomy.

Overall, 45.0% (*n* = 36) of women spontaneously achieved clinical pregnancy at a median of 4 months (IQR, 3–7) after the postoperative period, in which pregnancy was not permitted. In a binary logistic regression model (Table 1), the following two parameters were significantly associated with clinical pregnancy in the univariable and the multivariable model: patient age (adjusted OR 0.718, 95% CI 0.608–0.849; *p* > 0.001) and the sum of fibroid size (OR 1.245, 95% CI 1.072–1.446; *p* = 0.004). BMI, type of infertility, type of surgery, and maximum fibroid size had no effect on the association.

**Table 1:** Patient and fibroid characteristics and associations with clinical pregnancy estimated as odds ratio (OR) with 95% confidence interval

	Pregnancy achieved	Pregnancy not achieved	Univariate analysis		Adjusted analysis	
	*n* = 36	*n* = 44	Crude OR (95%CI)	*p* value	Adjusted OR (95%CI)	*p* value
Age (years), median (IQR))	32.4 (28.1;35.6)	36.7 (33.7;38.4)	0.766 (0.667;0.880)	< 0.001	0.718 (0.608;0.849)	< 0.001
BMI (kg/m^2^), median (IQR)	23.1 (20.6;25.4)	24.4 (21.8;28.2)	0.926 (0.817;1.050)	0.230	–	–
Secondary infertility, *n* (%)	14 (38.9)	24 (54.5)	0.530 (0.217;1.298)	0.165	–	–
Laparotomy, *n* (%)	16 (44.4)	13 (29.5)	1.908 (0.758;4.800)	0.170	–	–
Number of fibroids, *n* (%)						
1	18 (50.0)	28 (63.6)	Reference	0.472	–	–
2	11 (30.6)	10 (22.7)	1.711 (0.604;4.847)		–	
3	7 (19.4)	6 (13.6)	1.185 (0.525;6.726)		–	
Largest fibroid size (cm), median (IQR)	6.3 (5.0;8.9)	5.0 (4.0;8.0)	1.175 (0.995;1.388)	0.057	–	–
Sum of fibroid size (cm), median (IQR)	8.5 (6.0;11.8)	7.3 (4.1;9.0)	1.152 (1.021;1.299)	0.021	1.245 (1.072;1.446)	0.004

**Fig. 1:**
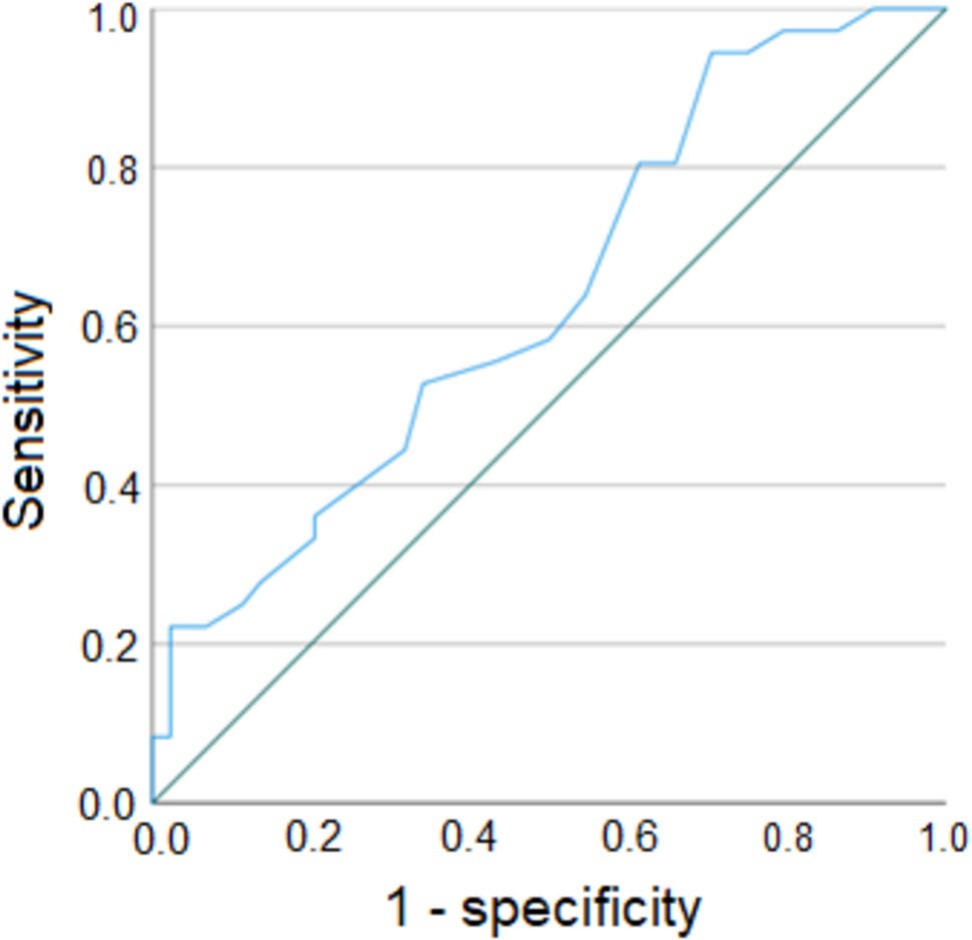
ROC curve for the sum of fibroid size and clinical pregnancy

**Fig. 2:**
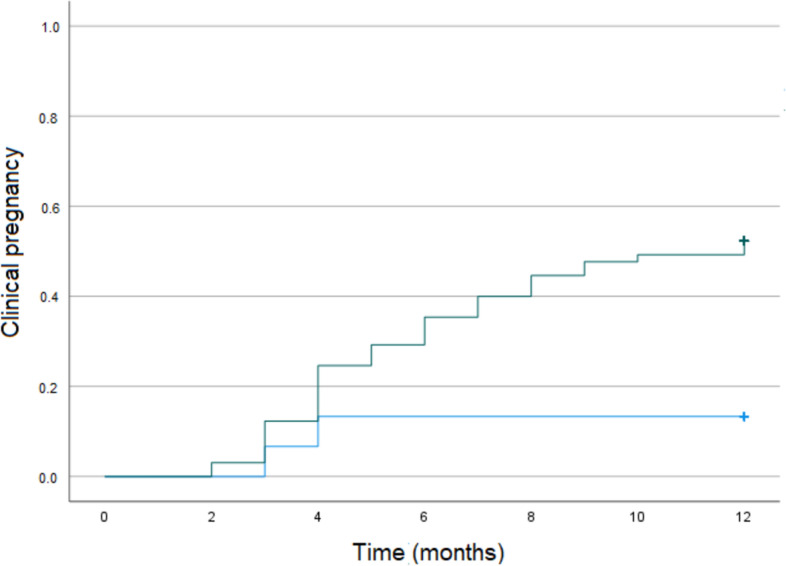
Kaplan–Meier curve for clinical pregnancy according to the sum of fibroid size (≥ 5 cm: gray line versus < 5 cm: blue line). Clinical pregnancy rates quadruples when removal of FIGO V and VI fibroids with a combined size greater than 5 cm compared to those with smaller fibroids

Since the sum of fibroid size was a significant parameter for clinical pregnancy, its optimized cut-off value was calculated. The according ROC curve is shown in Fig. 1. The maximum sum of sensitivity and specificity for pregnancy was at a sum of fibroid diameter of ≥ 5 cm (*p* = 0.008). Using this cut-off, the sensitivity was 94.4% (95% CI 81.3–99.3), whereas the specificity was 29.5% (95% CI 16.8–45.2). Thirty-four/65 patients with a sum of fibroid size ≥ 5 cm became pregnant (positive predictive value, PPV, 52.3%, 95% CI 39.6–98.3%), whereas thirteen/15 women with a sum of fibroid size < 5 cm did not achieve a pregnancy (negative predictive value, NPV, 86.7%, 95% CI 59.5–98.3%). The according Kaplan–Meier curve is shown in Fig. 2.

## Discussion

We present a cohort of 80 infertile women of childbearing age, who underwent laparoscopy or laparotomy for the removal of subserosal FIGO V and VI fibroids. While postoperative pregnancy outcomes for submucosal FIGO 0–3 fibroids are well studied and clear treatment recommendations are available, there are limited data on pregnancy rates following surgical removal of subserosal fibroids. The prevailing literature suggests that fibroids that distort the cavity (classified as International Federation of Gynecology and Obstetrics [FIGO] types 0–3 [21]) have a greater impact on fertility, with surgical interventions mostly restoring fecundity [22]. For all other fibroids, however, there is controversy. The existing literature is imprecise, contradictory, and of low quality [23].

In our study cohort of infertile women with subserous FIGO 5 and 6 fibroids, we observed an unassisted pregnancy rate of 45% within 12 months after non-hysteroscopic surgery. The postoperative odds of clinical pregnancy were higher in younger women and in women with larger fibroids (Table 1). The significant association between increasing age and decreasing pregnancy rates is well known and in accordance with preexisting data [24, 25]. However, the fact that women with a higher sum of fibroid size were more likely to benefit from myomectomy in terms of clinical pregnancy rates seems relevant and rather new. Some data suggest that leiomyomas > 2.85 cm [13] and > 3.0 cm [12], respectively, negatively impact conception, whereas other studies report clinical significance only in fibroids exceeding 5 cm. In our patient population, the latter could be confirmed as the maximum sum of sensitivity and specificity for pregnancy was at a sum of fibroid diameter of ≥ 5 cm (*p* = 0.008). Finding optimal sensitivity and specificity through an ROC should not be interpreted as fibroids only of a fixed size having an impact (a threshold). In contrast, the larger they are, the more probable seems their negative effect. Taking these findings a step further, where enucleation of smaller fibroids did not restore fertility, one of the most important takeaways should be that thorough exploration for other superimposed etiologies for subfertility is critical when the combined fibroid diameter is < 5 cm, since fecundity is less likely to be restored through myomectomy. However, our clear, direct finding is that myomectomy should be considered when the combined fibroid size is ≥ 5 cm, because it quadruples fecundability relative to those having removal of smaller fibroids. Specifically, only 13.3% of patients with a sum of fibroid size < 5 cm achieved clinical pregnancy compared to 52.3% of patients with a sum of fibroid size ≥ 5 cm. Myomectomy for FIGO V and VI fibroids for infertility should be considered for women when the size exceeds 5 cm, even though future studies will hopefully provide additional insight.

Several limitations must be acknowledged. As is common to similar studies, we were unable to record the number of live births or miscarriages. With the goal being live birth and not just conception, future research ideally should capture live birth data. We should also acknowledge that the best metric for fibroid burden is volume or mass of fibroid removed. Neither of these parameters were available. In addition, the retrospective study design is susceptible to confounding potentially limiting generalizability. However, the currently available evidence on pregnancy rates after non-hysteroscopic surgery for subserosal fibroids is based exclusively on small observational studies that included heterogeneous patient populations, used non-standardized fibroid classifications, and described fibroids only vaguely (e.g., location, size, and number of fibroids). We lowered this heterogeneity for our sample by including only FIGO-V and FIGO-VI fibroids and excluding patients over 40 years of age, reducing the impact of oocyte aneuploidy as a superimposed ovarian factor.

Regarding study strengths, we determined the clinically relevant parameter “time to pregnancy”. Moreover, strict exclusion criteria were applied. All women/couples with other causes for sub-/infertility were excluded. Furthermore, most existing publications blur the mode of conception postoperatively. We report an unassisted pregnancy rate of 45% in our population. Last but not least, an advantage of our study is the homogeneity of fibroid location (FIGO V and FIGO VI) that supports clear clinical recommendations, where almost 50% of all infertile women aged 18–40 years became pregnant within 24 months following the surgical removal of FIGO V and FIGO VI fibroids via laparoscopy or laparotomy.

## Conclusion

Women with a combined sum of fibroid diameters ≥ 5 cm are four times more likely to spontaneously conceive after myomectomy than similar women with smaller fibroids. These findings support a dose–response effect for how we look at fibroids, where the impact of pathology is likely probabilistic and on a continuum. Not only do the results support myomectomy for larger type V and VI fibroids, but also they speak to the importance of a more thorough workup for other factors in women with smaller type V and VI fibroids and otherwise unexplained infertility, since they are far less likely to benefit from surgical intervention.

## Data Availability

Data is provided upon reasonable request.

## References

[1] Baird DD, Dunson DB, Hill MC, Cousins D, Schectman JM (2003) High cumulative incidence of uterine leiomyoma in black and white women: ultrasound evidence. Am J Obstet Gynecol 188(1):100–107 12548202 10.1067/mob.2003.99

[2] Stewart EA, Cookson CL, Gandolfo RA, Schulze-Rath R (2017) Epidemiology of uterine fibroids: a systematic review. BJOG 124(10):1501–1512 28296146 10.1111/1471-0528.14640

[3] Bosteels J, van Wessel S, Weyers S, Broekmans FJ, D’Hooghe TM, Bongers MY et al (2018) Hysteroscopy for treating subfertility associated with suspected major uterine cavity abnormalities. Cochrane Database Syst Rev. 10.1002/14651858.CD009461.pub4 30521679 10.1002/14651858.CD009461.pub4 PMC6517267

[4] Khaund A, Lumsden MA (2008) Impact of fibroids on reproductive function. Best Pract Res Clin Obstet Gynaecol 22(4):749–760 18547868 10.1016/j.bpobgyn.2008.01.009

[5] Rackow BW, Taylor HS (2010) Submucosal uterine leiomyomas have a global effect on molecular determinants of endometrial receptivity. Fertil Steril 93(6):2027–2034 18555231 10.1016/j.fertnstert.2008.03.029 PMC3107853

[6] Guo XC, Segars JH (2012) The impact and management of fibroids for fertility: an evidence-based approach. Obstet Gynecol Clin North Am 39(4):521–533 23182558 10.1016/j.ogc.2012.09.005 PMC3608270

[7] Don EE, Middelkoop MA, Hehenkamp WJK, Mijatovic V, Griffioen AW, Huirne JAF (2023) Endometrial angiogenesis of abnormal uterine bleeding and infertility in patients with uterine fibroids-a systematic review. Int J Mol Sci 24(8):7011 37108180 10.3390/ijms24087011 PMC10138959

[8] Horne AW, Critchley HO (2007) The effect of uterine fibroids on embryo implantation. Semin Reprod Med 25(6):483–489 17960533 10.1055/s-2007-991046

[9] Agostinis C, Mangogna A, Bossi F, Ricci G, Kishore U, Bulla R (2019) Uterine immunity and microbiota: a shifting paradigm. Front Immunol 10:2387 31681281 10.3389/fimmu.2019.02387 PMC6811518

[10] Roy KK, Singla S, Baruah J, Sharma JB, Kumar S, Singh N (2010) Reproductive outcome following hysteroscopic myomectomy in patients with infertility and recurrent abortions. Arch Gynecol Obstet 282(5):553–560 20512650 10.1007/s00404-010-1531-0

[11] Shokeir T, El-Shafei M, Yousef H, Allam AF, Sadek E (2010) Submucous myomas and their implications in the pregnancy rates of patients with otherwise unexplained primary infertility undergoing hysteroscopic myomectomy: a randomized matched control study. Fertil Steril 94(2):724–729 19406399 10.1016/j.fertnstert.2009.03.075

[12] Christopoulos G, Vlismas A, Salim R, Islam R, Trew G, Lavery S (2017) Fibroids that do not distort the uterine cavity and IVF success rates: an observational study using extensive matching criteria. BJOG 124(4):615–621 27921379 10.1111/1471-0528.14362

[13] Yan L, Ding L, Li C, Wang Y, Tang R, Chen ZJ (2014) Effect of fibroids not distorting the endometrial cavity on the outcome of in vitro fertilization treatment: a retrospective cohort study. Fertil Steril 101(3):716–721 24424367 10.1016/j.fertnstert.2013.11.023

[14] Horcajadas JA, Goyri E, Higon MA, Martinez-Conejero JA, Gambadauro P, Garcia G et al (2008) Endometrial receptivity and implantation are not affected by the presence of uterine intramural leiomyomas: a clinical and functional genomics analysis. J Clin Endocrinol Metab 93(9):3490–3498 18559911 10.1210/jc.2008-0565

[15] Johnson G, MacLehose RF, Baird DD, Laughlin-Tommaso SK, Hartmann KE (2012) Uterine leiomyomata and fecundability in the right from the start study. Hum Reprod 27(10):2991–2997 22811308 10.1093/humrep/des263 PMC3442631

[16] Goldberg HR, McCaffrey C, Amjad H, Kives S (2022) Fertility and pregnancy outcomes after robotic-assisted laparoscopic myomectomy in a Canadian cohort. J Minim Invasive Gynecol 29(1):72–76 34192566 10.1016/j.jmig.2021.06.015

[17] Yoshino O, Nishii O, Osuga Y, Asada H, Okuda S, Orisaka M et al (2012) Myomectomy decreases abnormal uterine peristalsis and increases pregnancy rate. J Minim Invasive Gynecol 19(1):63–67 22070929 10.1016/j.jmig.2011.09.010

[18] Organization WH. Infertility 2024 [Available from: https://www.who.int/news-room/fact-sheets/detail/infertility. Accessed 30 Nov 2024

[19] Mais V, Ajossa S, Guerriero S, Mascia M, Solla E, Melis GB (1996) Laparoscopic versus abdominal myomectomy: a prospective, randomized trial to evaluate benefits in early outcome. Am J Obstet Gynecol 174(2):654–658 8623802 10.1016/s0002-9378(96)70445-3

[20] Holzer A, Jirecek ST, Illievich UM, Huber J, Wenzl RJ (2006) Laparoscopic versus open myomectomy: a double-blind study to evaluate postoperative pain. Anesth Analg 102(5):1480–1484 16632830 10.1213/01.ane.0000204321.85599.0d

[21] Munro MG, Critchley HO, Broder MS, Fraser IS, Disorders FWGoM (2011) FIGO classification system (PALM-COEIN) for causes of abnormal uterine bleeding in nongravid women of reproductive age. Int J Gynaecol Obstet 113(1):3–13 21345435 10.1016/j.ijgo.2010.11.011

[22] Practice Committee of the American Society for Reproductive Medicine, Electronic address Aao, Practice Committee of the American Society for Reproductive M (2017) Removal of myomas in asymptomatic patients to improve fertility and/or reduce miscarriage rate: a guideline. Fertil Steril 108(3):416–425 28865538 10.1016/j.fertnstert.2017.06.034

[23] Metwally M, Raybould G, Cheong YC, Horne AW (2020) Surgical treatment of fibroids for subfertility. Cochrane Database Syst Rev. 10.1002/14651858.CD003857.pub4 31995657 10.1002/14651858.CD003857.pub4 PMC6989141

[24] Hartmann KE, Velez Edwards DR, Savitz DA, Jonsson-Funk ML, Wu P, Sundermann AC et al (2017) Prospective cohort study of uterine fibroids and miscarriage risk. Am J Epidemiol 186(10):1140–1148 28591761 10.1093/aje/kwx062 PMC5860279

[25] Sundermann AC, Velez Edwards DR, Bray MJ, Jones SH, Latham SM, Hartmann KE (2017) Leiomyomas in pregnancy and spontaneous abortion: a systematic review and meta-analysis. Obstet Gynecol 130(5):1065–1072 29016496 10.1097/AOG.0000000000002313 PMC5656535

